# Treating Problem Drinking

**Published:** 1999

**Authors:** Kimberly S. Walitzer, Gerard J. Connors

**Affiliations:** Kimberly S. Walitzer, Ph.D., is deputy director and Gerard J. Connors, Ph.D., is director of the Research Institute on Addictions, Buffalo, New York

**Keywords:** problematic AOD (alcohol and other drug) use, disease severity, cognitive therapy, behavior therapy, biological AOD dependence, controlled AOD use, AODU (alcohol and other drug use) treatment method, AOD abstinence, amount of AOD use, AOD use frequency, treatment outcome, predictive factor, gender differences, motivation, literature review

## Abstract

Recent data suggest that most people experiencing alcohol problems have problems of mild to moderate severity. Relative to alcoholics, these drinkers have a shorter problem-drinking history, greater social and economic stability, and greater personal resources. This article describes a cognitive-behavioral treatment approach designed specifically for problem drinkers with low levels of physical dependence on alcohol who choose to reduce their drinking. After describing various drinking-reduction techniques, the article reviews empirical evidence for drinking-reduction training. The increasing availability of drinking-reduction interventions holds considerable promise for reducing alcohol-related dysfunction among problem drinkers.

The review of epidemiological literature on alcohol abuse in the *Ninth Special Report to the U.S. Congress on Alcohol and Health* (National Institute on Alcohol Abuse and Alcoholism 1997) suggests that alcohol use in the United States has been declining for almost two decades. For example, the prevalence of current drinkers (i.e., those consuming alcohol in the previous year) decreased from 70 percent in 1984 to 65 percent in 1990; weekly drinking decreased from 36 percent to 29 percent, and “heavy” drinking (defined as consuming five or more drinks^1^ on one occasion at least weekly) decreased from 6 percent to 4 percent during the same period. The same review, however, also indicates that the prevalence of social consequences (e.g., problems with family, fighting, and financial problems) and dependence symptoms (e.g., binge drinking and loss of control over drinking) associated with drinking has not shown a corresponding decline. For example, three or more alcohol-dependence symptoms were reported by 7 percent of current drinkers in 1984 and by 8 percent of current drinkers in 1990; reports of two or more social consequences increased from 11 percent to 13 percent during that period. In sum, data cited in the *Ninth Special Report* indicate that although several indices of alcohol consumption have evidenced a decline in recent years, dependence symptoms and social consequences from drinking have been stable to increasing.

Data from a 1992 survey of the general population (Caetano 1997) suggest that most people experiencing alcohol problems have problems of mild to moderate severity. Of the people who reported at least one dependence-related problem or social consequence, few experienced more than two such problems. The data are consistent across demographic lines, including black, white, and Hispanic men and women, suggesting that occasional and mild-to-moderate alcohol problems represent the most prevalent form of alcohol problems. Caetano’s research also indicates a significant number of male and female drinkers who are unlikely to meet diagnostic criteria for severe alcohol dependence or alcoholism, despite their frequent drinking and occasional negative consequences from drinking. Rather, the majority of this group are more appropriately classified as problem drinkers.

## Defining Problem Drinking

Although the terms “problem drinking” and “alcoholism” sometimes are used interchangeably, researchers in the alcohol field have distinguished between the two concepts. Sobell and Sobell’s (1993) review of the literature suggests that relative to alcoholics, problem drinkers have a shorter problem-drinking history, greater social and economic stability, and greater personal resources. They typically have not experienced major losses because of their drinking and have not exhibited severe withdrawal symptoms, such as seizure or delirium tremens, upon cessation of past drinking. This article uses the term “problem drinking” to describe people who drink heavily or experience occasional problems from drinking but who do not have a history of severe physical dependence on alcohol.^2^

Many problem drinkers choose to reduce their drinking rather than pursue abstinence as a solution to their concerns about drinking. In response, drinking-reduction approaches have been developed to help problem drinkers reduce alcohol consumption and minimize risks associated with their drinking (Rosenberg 1993). Abstinence-based treatment strategies that have been empirically evaluated with alcoholic populations are easily and minimally adapted to meet the needs of problem drinkers who choose to pursue a goal of abstinence. Because those abstinence-based treatment strategies are described elsewhere in this issue, this article focuses on treatment approaches designed specifically for problem drinkers with low levels of physical dependence on alcohol who choose to reduce their drinking. After describing various drinking-reduction techniques, the article also reviews empirical evidence for drinking-reduction training.

## Strategies for Treatment of Problem Drinking

For problem drinkers, the first issue of treatment is to choose either an abstinence goal or a moderation goal (see “Issues Regarding Goal Choice” later in this article). This consideration may take into account the history of the drinker, an assessment of physical dependence on alcohol, and any medical or other drawbacks to moderate alcohol use (see Sanchez-Craig [1993] for an example of this decisionmaking process). For problem drinkers pursuing a goal of moderation, a cognitive-behavioral approach may include a variety of strategies (e.g., Hester 1995; Miller and Munoz 1982; Sanchez-Craig 1993; Sobell and Sobell 1993), such as self-monitoring; goal setting; functional analysis of drinking behavior; learning to respond differently to situations that are associated with drinking, such as walking past one’s favorite bar (i.e., stimulus-control training); and strategies for modifying drinking behavior (e.g., decreasing sip rate and amount, preplanning drinking, and setting drinking limits). Drinkers are presented with techniques and strategies to heighten their awareness of drinking and to reduce drinking and associated risks.

To be able to monitor and evaluate changes in drinking, problem drinkers first must be aware of the quantity of alcohol they drink and any relevant drinking patterns. Thus, two central components of the drinking-moderation approach are daily self-monitoring of alcohol consumption and goal setting. Self-monitoring may consist of simply recording the number of standard drinks consumed for each drinking occasion and calculating weekly totals, or it also may include monitoring the duration of drinking; drinking situations, companions, and consequences; and use of reduction strategies.

The second fundamental strategy in drinking-moderation training is the setting of specific drinking-reduction goals. Ideally, drinking-reduction goals are selected by the drinker to maximize motivation, personal control, and responsibility, although “safe” drinking guidelines are also typically provided for the drinker to take into consideration. Goals typically include a maximum number of standard drinks per week, day, and drinking occasion as well as the desired frequency of abstinent days per week. More detailed goals, based on the individual needs of the drinker, may include no drinking or limited drinking during high-risk situations (e.g., when angry, with certain people, or before driving), a maximum drinking rate (e.g., no more than one standard drink per hour), and eliminating or changing beverage type or strength (e.g., no shots, no multiple-shot mixed drinks, or changing to reduced-potency beverages). For drinking-reduction goals to be feasible, it may be necessary for the drinker to set a series of short-term drinking-reduction goals, such that attaining each short-term goal brings the drinker closer to his or her long-term goals. Although specific moderation goals will vary for each drinker, the overarching goal for all drinkers is to reduce alcohol consumption and, when alcohol is consumed, to minimize alcohol-related risks.

With self-monitoring and goal setting as a foundation, drinkers learn and practice a variety of cognitive-behavioral strategies (see Miller and Munoz 1982; Sanchez-Craig 1993; Sobell and Sobell 1993) for changing drinking patterns. These strategies include methods for reducing drinking (e.g., decreasing sip amount and sip rate, spacing drinks, and alternating nonalcoholic beverages with alcoholic beverages) and eliminating drinking (e.g., substituting a nonalcoholic beverage during what would otherwise be a drinking occasion or not drinking [or delaying drinking] in the presence of a strongly associated drinking stimulus). Other strategies focus on enhancing self-preparation, self-control, and motivation (e.g., planning ahead to limit drinking during a future drinking occasion, positive self-talk, self-rewards, and acknowledgment of effort).

With regard to gauging treatment response, it is difficult to provide specific guidelines that are appropriate for all problem drinkers. Instead, response to treatment is most appropriately assessed by the client and therapist in the context of the drinker’s pretreatment drinking history and individualized alcohol-consumption goals. Outcome may be conceptualized in terms of drinking frequency (e.g., number of abstinent days per week), drinking quantity (e.g., number of “light” drinking days per week), a weekly goal (e.g., drinking less than a weekly consumption limit), lack of negative consequences from drinking and, perhaps most important, lowered risk for future problems from drinking. The authors’ clinical experience suggests that some clients who are unable to reduce their drinking and their risk for drinking-related problems after 2 to 3 months of focused treatment become amenable to more intensive and abstinence-based treatment.

### Empirical Evidence Supporting Drinking-Reduction Training

Empirical evaluation of the effectiveness of drinking-reduction training for problem drinkers has taken place during the past several decades. One program of research by Miller and colleagues at the University of New Mexico involved a long-term followup of four samples of problem drinkers who received moderation treatment based on goal-setting and self-monitoring strategies (Miller et al. 1992). Outcome, measured at time points ranging from 3.5 years to 8 years following treatment, was assessed for 71 percent of the participants.^3^ At followup, 23 percent were classified as abstinent and 14 percent were classified as asymptomatic drinkers (i.e., had no drinking-related problems in the period preceding the followup); 22 percent were improved but reported at least two problems in the previous year, and 35 percent of the sample were unremitted (i.e., were experiencing alcohol-related problems at a level comparable to or greater than when they entered the study). Similar outcomes have been reported by a variety of investigators (e.g., Alden 1988; Brown 1980; Connors et al. 1992; Sanchez-Craig et al. 1984). Taken together, these studies demonstrate the robust efficacy of behavioral self-control strategies in reducing alcohol consumption, even with variations in technique and different samples.

### Issues Regarding Goal Choice

Sanchez-Craig and her colleagues (Sanchez-Craig 1980; Sanchez-Craig et al. 1984) have evaluated the influence on treatment of clients’ preference of a moderate-drinking goal relative to an abstinence goal. In one study, in which clients were randomly assigned to an abstinence versus moderate-drinking goal, clients assigned to the abstinence goal drank more frequently and in a greater quantity during treatment compared with those assigned to a moderate-drinking goal; furthermore, they developed moderate-drinking patterns on their own by the second-year followup despite the abstinence-based treatment (Sanchez-Craig et al. 1984). In a second study, when problem drinkers were given the choice of abstinence or a moderate-drinking goal, 80 percent chose a goal of moderate drinking (Sanchez-Craig et al. 1989). Reduced drinking, relative to abstinence, appears to be an effective as well as palatable goal for problem drinkers.

### Predictors of Success

Although no single, strong predictor of successful response to drinking-moderation treatments has been identified, several client characteristics frequently emerge as having some predictive value (Rosenberg 1993). In this regard, two characteristics that are useful in identifying people likely to be successful with a drinking-reduction goal are lessened severity of physical dependence on alcohol and the drinker’s belief that drinking moderation is attainable. The authors’ clinical research in this area uses additional eligibility criteria, including absence of current, recent, or multiple alcohol-related legal offenses; absence of alcohol-related hospitalizations, including detoxification or inpatient treatment; absence of medical contraindications to moderate alcohol consumption (e.g., impaired liver function or pregnancy); and being of legal drinking age.

### Gender Differences

Drinking-reduction interventions appear to have particularly positive benefits among problem-drinking women. Three reports (Miller and Joyce 1979; Sanchez-Craig et al. 1989, 1996) have shown that women problem drinkers were even more successful in attaining moderate drinking than were men. In the Sanchez-Craig and colleagues (1996) study, for example, more women than men (71 percent versus 52 percent) were classified as moderate drinkers at the 12-month followup. These reports indicate that relative to their male counterparts, moderate-drinking interventions may be especially effective for problem-drinking women.

## Modifications and Enhancements to Treatment

As the previous review has indicated, empirical research on cognitive-behavioral drinking-reduction approaches indicates that these strategies are useful in helping problem drinkers reduce their alcohol consumption. Research has examined the effectiveness of modifying drinking-moderation training to bibliotherapy^4^ and telephone therapy formats. Also, because the studies noted earlier reveal that some drinkers (one-third to one-half, depending on the study) remain heavy drinkers following treatment (e.g., Miller et al. 1992), researchers have attempted to enhance treatment outcome with additional treatment components. The following sections review several efforts to modify basic drinking-reduction treatment and enhance treatment outcome.

### Bibliotherapy

Since 1980, 10 studies have evaluated the use of bibliotherapy as a drinking-reduction intervention with problem drinkers. The self-help books used in these studies typically have included instructions on self-monitoring, goal-setting, and drinking-reduction strategies (e.g., Sanchez-Craig 1993).

In 5 of the 10 studies using bibliotherapy to reduce alcohol consumption, bibliotherapy was compared with more intensive, therapist-directed treatment. In four of the five studies (Miller and Baca 1983; Miller and Taylor 1980; Miller et al. 1981; Skutle and Berg 1987), a behavioral self-help manual was used as a bibliotherapy or as part of an intervention involving therapist contacts and/or additional skills-building treatment components (e.g., relaxation techniques or assertion skills). In each case, subjects showed equal and significant reductions on a variety of drinking-related variables. The fifth study (Harris and Miller 1990) included a waiting-list control group that engaged in self-monitoring of drinking and showed significant reductions in alcohol consumption among subjects who received either self-directed behavioral self-control training (i.e., bibliotherapy) or therapist-directed self-control training. Both the bibliotherapy group and the therapist-directed group showed greater reductions in drinking-related variables than did subjects in the control group.

In four other studies, client use of a self-help manual and/or materials on how to reduce drinking was compared with receipt of general advice and/or information on alcohol effects. In each case, persons receiving the self-help materials had superior outcomes to persons receiving the general information packets (Heather et al. 1987, 1990; Sitharthan et al. 1996; Spivak et al. 1994).

Finally, in the 10th study, Sanchez-Craig and colleagues (1996) mailed self-identified problem drinkers a self-help book and either did or did not supplement this book with a 30-minute telephone assessment that included feedback and a motivational interview. Clients receiving the feedback/motivational interview session had better outcomes at the 3-month followup, but at 12 months, the two groups had comparable positive outcomes.

In a related study, Hester and Delaney (1997) examined the use of a computer-based, interactive adaptation of behavioral self-control strategies, rather than the booklet and self-help materials previously described. Participants who completed the eight-session, self-directed computer program reported significantly reduced drinking relative to a waiting-list control group. The authors noted several potential advantages of computer-assisted self-help materials, including the ability to customize the program on the basis of pretreatment assessment and to provide individualized graphic feedback on progress.

### Telephone Therapy

The administration of interventions via the telephone has been used for many years as an adjunct to the traditional provision of health services (e.g., to handle crises, provide additional support, and answer questions). As described earlier, Sanchez-Craig and colleagues (1996) included a telephone intervention in their study of treating problem drinking with bibliotherapy. Clients received a self-help book by mail and either received or did not receive a 30-minute assessment feedback/motivational interview session. Compared with clients who did not receive the feedback/motivational interview session, significantly more clients who received the session were classified as moderate drinkers at 3-month followup, although at 12 months both groups of clients were doing equally well.

### Motivational Approaches

Several evaluations of drinking-moderation interventions (e.g., Sanchez-Craig et al. 1996; Skutle and Berg 1987; Sobell et al. 1996) have incorporated components of motivational interviewing as strategies for engaging people in the change process. In discussing this issue, Miller and Rollnick (1991) suggested that motivation can be defined as “the probability that a person will enter into, continue, and adhere to a specific change strategy” (p. 19). From this perspective, intervening with the client to enhance motivation seems like a natural approach for increasing the likelihood of engaging in drinking-reduction treatment. Brown and Miller (1993) found that patients who received a two-session motivational assessment and interview shortly after intake to a residential alcoholism treatment program participated more fully in treatment and consumed less alcohol at 3-month followup than did patients who received no motivational intervention. Bien and colleagues (1993) examined the effectiveness of a motivational intervention in preparation for a Veterans Affairs outpatient substance abuse treatment program. Patients who received the motivational interview had better alcohol outcomes at 3-month followup than did control participants, although by the 6-month followup, group differences were no longer significant. Taken together, the evidence suggests that a motivational-interviewing approach has considerable promise for facilitating positive outcomes in drinking-reduction interventions.

### Other Modifications

Three additional modifications and enhancements to drinking-reduction treatment are under investigation in the authors’ research: a program specifically designed for women, a program that includes involvement of the drinker’s spouse, and an outreach program for heavy drinkers in rural areas. The rationale for and the treatment components of each of these programs are described briefly in the paragraphs that follow.

The research reviewed earlier in this article indicates that a number of women experience alcohol-related problems and that interventions focused on drinking reduction appear to be especially effective for heavily drinking women. One research effort in this area (Connors and Walitzer 1997) has focused on developing and assessing interventions specifically geared toward women drinkers. In the “Women and Health Program,” self-referred problem-drinking women without histories of severe physical dependence on alcohol received a 10-week behavioral self-control treatment focusing on alcohol reduction. The treatment emphasized increasing abstinent and light-drinking days and decreasing heavy-drinking days. The effects of two treatment enhancements—7 hours of training in life-management skills (i.e., problem-solving, communication, assertiveness, and other skills) and a series of eight booster sessions—were assessed during the 18 months following treatment. The life-management skills were designed to enhance social, coping, and other skills to maximally equip the women to address psychosocial and situational antecedents to their problem drinking. The booster sessions, which occurred at lengthening intervals over the 6 months following treatment, were designed to reinforce the material presented during treatment.

Spouse-involved treatment has been investigated as a treatment enhancement for alcohol-dependent populations (e.g., McCrady et al. 1991; O’Farrell et al. 1992). Involving the spouse in alcoholism treatment has been found to produce better outcomes than has individual treatment that excludes the spouse (e.g., Bowers and Al-Redha 1990; McCrady et al. 1991). Spouse-involved therapy can remain focused on the alcohol problem and the spouse’s appropriate role in supporting the client, or it can include direct attempts to improve marital functioning through behavioral marital therapy—a well-researched and effective technique for alleviating marital distress (e.g., Hahlweg and Markman 1988). Walitzer and Connors are conducting a research program that focuses on heavy drinkers and their spouses, the “Couples Drinking Reduction Program.” The program evaluates the additive effects of teaching spousal support of drinking moderation and of behavioral marital therapy in a drinking-moderation program.

People living in rural areas—representing approximately 25 percent of the U.S. population—face a variety of barriers to health care services. Those barriers include geography, no or little insurance, poverty, and limited access to public transportation (Rodrigue et al. 1996). Furthermore, the scarcity of health care professionals and agencies complicates the delivery of health services to rural areas. In fact, two-thirds of rural Americans live in areas identified as having insufficient psychological and psychiatric services (Wagenfeld 1990). One approach to providing greater health care access to people in rural areas is to develop programs that reduce or avoid traditional barriers to service access. To that end, the authors are conducting a research-based rural outreach program that is examining bibliotherapy and telephone therapy interventions as strategies for helping rural heavy drinkers reduce alcohol consumption.

## Conclusions

Epidemiological research indicates that alcohol use is associated with negative consequences among significant numbers of drinkers and that most of those drinkers experience problems of mild to intermediate severity. For many problem drinkers, interventions focusing on drinking moderation are both palatable and effective. Indeed, some data (e.g., Sanchez-Craig 1980) suggest that moderate-drinking interventions should be viewed as the appropriate treatment for problem drinkers without histories of severe dependence on alcohol who desire to reduce their use of alcohol rather than abstain. Although cognitive-behavioral drinking-reduction techniques have become somewhat more available, they have not yet been widely disseminated in the professional treatment community. The self-help program Moderation Management (Kishline 1994), which focuses on the reduction of alcohol use and associated risks for problem drinkers, is becoming more widespread but has not yet been empirically studied. It is the authors’ hope that drinking-moderation techniques for problem drinkers will continue to be increasingly available and accepted as well as researched.

## Footnotes

1 Although the definition of a standard drink can vary from study to study, it is commonly defined as 12 ounces (oz) of beer, 5 oz of wine, or 1.5 oz of distilled spirits.

2 This definition is consistent with that proposed by the Institute of Medicine (1990) report, which defined problem drinkers as those having moderate to substantial levels of alcohol consumption and possibly alcohol abuse but who are unlikely to have physical dependence on alcohol.

3 *N* = 94; average age = 50.2 years; 45 percent of the sample was female.

4 The term “bibliotherapy” denotes the therapeutic use of written materials to effect behavioral change.
